# Value of [^18^F]-FDG positron emission tomography in patients with recurrent glioblastoma receiving bevacizumab

**DOI:** 10.1093/noajnl/vdaa050

**Published:** 2020-04-15

**Authors:** Maya S Graham, Simone Krebs, Tejus Bale, Kwaku Domfe, Stephanie M Lobaugh, Zhigang Zhang, Mark P Dunphy, Thomas Kaley, Robert J Young

**Affiliations:** 1 Department of Neurology, Memorial Sloan Kettering Cancer Center, New York, New York, USA; 2 Department of Radiology, Molecular Imaging and Therapy Service, Memorial Sloan Kettering Cancer Center, New York, New York, USA; 3 Department of Pathology, Memorial Sloan Kettering Cancer Center, New York, New York, USA; 4 College of Medicine, SUNY Upstate Medical University, Syracuse, New York, USA; 5 Department of Epidemiology and Biostatistics, Memorial Sloan Kettering Cancer Center, New York, New York, USA; 6 Department of Radiology, Neuroradiology Service, Memorial Sloan Kettering Cancer Center, New York, New York, USA; 7 The Brain Tumor Center, Memorial Sloan Kettering Cancer Center, New York, New York, USA

**Keywords:** bevacizumab, FDG, glioblastoma, positron emission tomography

## Abstract

**Background:**

Treatment of recurrent glioblastoma (GBM) with bevacizumab can induce MRI changes that confound the determination of progression. We sought to determine the value of [^18^F]-fluorodeoxyglucose (FDG) positron emission tomography (PET) in GBM patients receiving bevacizumab at the time of suspected progression and, thereby, its utility as a potential prognostic adjunct in progressive disease.

**Methods:**

This retrospective study included patients who underwent brain FDG PET within 4 weeks of receiving bevacizumab for recurrent GBM with suspected progression. Volumes-of-interest were placed over the reference lesion with measurement of maximum standardized uptake value (SUV_max_), peak standardized uptake value (SUV_peak_), metabolic tumor volume, total lesion glycolysis (TLG), and tumor-to-normal contralateral white matter ratios (TNR-WM). Tumors were additionally categorized as non-avid or avid based on qualitative FDG uptake. Associations between baseline variables and overall survival (OS) were examined using univariable and multivariable Cox proportional hazards regression, with *P* < .05 considered significant.

**Results:**

Thirty-one patients were analyzed. Qualitative FDG uptake was significantly associated with OS (*P* = .03), with a median OS of 9.0 months in non-avid patients versus 4.5 months in avid patients. SUV_max_, SUV_peak_, TNR-WM, and TLG were significantly associated with OS (*P* < .001, TLG: *P* = .009). FDG avidity and SUV_max_ remained significantly associated with OS (*P* = .046 and .048, respectively) in the multivariable analysis including age, KPS, and *MGMT* status. Dichotomizing patients using an SUV_max_ cutoff of 15.3 was associated with OS (adjusted *P* = .048).

**Conclusion:**

FDG PET is a promising imaging tool to further stratify prognosis in recurrent GBM patients on antiangiogenic therapy.

**Key Points**

- FDG PET is negatively associated with overall survival in recurrent GBM patients on bevacizumab, including chronic treatment.
- SUV_max_ can dichotomize these patients by overall survival.

**Importance of the Study**

The ability of bevacizumab to induce changes in contrast-enhanced MRI appearance that hamper determination of GBM progression is well known. Several recent studies have evaluated alternative radiographic measures of progressive disease in this setting, many of which are not widely available and with no clear consensus. To our knowledge, the present study is the first evaluation of the prognostic value of FDG PET at the time of suspected progression in recurrent GBM patients receiving antiangiogenic therapy. We found that FDG avidity was associated with inferior overall survival in these patients and observed an association with survival when patients were dichotomized by an optimal SUV_max_ cutoff. Importantly, our cohort included many patients on chronic bevacizumab, mirroring its typical clinical use. These results suggest the utility of FDG PET, a broadly accessible technique, as an adjunct in further stratifying prognosis in recurrent GBM patients on antiangiogenic therapy.

Glioblastoma (GBM) is the most common malignant primary brain tumor, and recurrence is virtually inevitable. A hallmark of the disease is an extensive network of rapidly growing and abnormally tortuous blood vessels stimulated by potent angiogenic signals including vascular endothelial growth factor (VEGF). Bevacizumab, a recombinant humanized monoclonal antibody targeting VEGF-A, is approved by the Food and Drug Administration (FDA) to treat recurrent GBM. Through its direct antiangiogenic actions, bevacizumab reduces tumor vasculature and permeability while promoting the normalization of remaining tumor vasculature. Because these changes may occur independent of actual antitumor effects, the decreased contrast enhancement on MRI may confound the detection of recurrent tumors.^1^ The unchanged tumor viability is supported by the lack of convincing improvement in overall survival (OS) with the addition of bevacizumab in recurrent GBM despite enhanced radiographic response rates.^2^

To address this limitation of conventional contrast-enhanced MRI in assessing tumor viability in the context of antiangiogenic therapy, the Response Assessment in Neuro-Oncology (RANO) group suggested the incorporation of T2 or FLAIR signal hyperintensity as an ancillary indicator of treatment response independent of contrast enhancement.^3^ However, a multitude of processes beyond non-enhancing tumor progression, such as radiation injury, peritumoral edema, or ischemia, can also lead to an increase in T2/FLAIR signal hyperintensity.^4^ Moreover, there is no consensus provided as to a quantitative degree of increase in the T2/FLAIR signal that constitutes progression. Several alternative imaging techniques have thus been proposed to assess progressive disease, including perfusion imaging,^5–8^ diffusion-weighted imaging,^9,10^ radiomics,^11,12^ and metabolic imaging with different positron emission tomography (PET) radiotracers.^13–15^

While several of these approaches have shown some preliminary promise, their overwhelming focus has been on early assessment within the first month of antiangiogenic therapy. The median progression-free survival (PFS) in recurrent GBM patients on bevacizumab is approximately 4 months,^2^ however, and few studies have examined the utility of ancillary imaging techniques in detecting GBM progression during such prolonged treatment with bevacizumab.^16^ In addition, many of the explored techniques require specialized amino acid radiotracers or complex radiographic analyses that are not widely available.^18^F-Fluorodeoxyglucose (FDG) PET represents an accessible and FDA-approved imaging modality that has been used to differentiate radiation necrosis from tumor recurrence of enhancing brain lesions on MRI, distinguish glioma from CNS lymphoma, and diagnose opportunistic infections.^17^ In this retrospective study, we evaluated the prognostic value of FDG PET imaging at the time of suspected progression in recurrent GBM patients on bevacizumab.

## Materials and Methods

### Patient Selection

This study was approved by the local institutional review board with a waiver of informed consent. Participants were retrospectively identified according to the following criteria: (1) pathological diagnosis of WHO grade IV GBM, (2) pathological or clinical/radiographic diagnosis of recurrence, (3) age at least 18 years, (4) no known mutation in *IDH1* or *IDH2*, and (5) imaged with^18^F-FDG PET while on bevacizumab therapy (defined as ≤4 weeks from the last dose) between January 2009 and January 2019. PET scans were performed as part of routine clinical care at the time of suspected progression based on RANO criteria. An experienced neuro-oncologist extracted patient data from hospital medical records including demographics, tumor and treatment characteristics, and OS. In this study, OS was defined as the time from the date of the FDG PET scan to the date of death (*n* = 29) or last follow-up (*n* = 2). Patients treated with bevacizumab at an initial diagnosis under NCT00782756^16^ were excluded from the analysis.

### ^18^F-Fluorodeoxyglucose PET/CT Protocol

Before the injection of ^18^F-FDG, all patients were required to fast for at least 6 h. If the plasma glucose level was less than 200 mg/dL, the patient was injected intravenously with 370 MBq of a radiotracer. After approximately 60–90 min uptake time, patients were scanned while in the supine position on PET/CT scanners (GE Discovery series VCT, 690, 710, GE Healthcare). Cross-calibration between the dose calibrator and PET scanners was performed monthly. Low-dose CT images obtained during PET/CT were used for attenuation correction of the PET emission scan and for anatomical orientation. PET/CT images were reconstructed using an ordered-subsets expectation maximization algorithm and a Gaussian filter using the standard manufacturer-supplied reconstruction software. The acquisition and reconstruction parameters were harmonized to minimize differences in standardized uptake values (SUVs) between scanners and keep them within 10%, as tested using measurements of the IEC image quality phantom. A spiral CT was acquired using a full helical acquisition at 1 s/rotation,150 mA, 120–140 kV; slice thickness, 3.75 mm. Immediately upon completion of the CT, a 10-min 3D PET scan was acquired. CT and PET data were reconstructed using a 30-cm field of view.

### Image Interpretation, Lesion Detection, and Data Analysis

A nuclear medicine physician defined 3-dimensional volumes-of-interest (VOIs) for the lesion and normal centrum semiovale white matter on a GE Advantage workstation using the PET VCAR application of the Volume Viewer software package (v. 12.3 Ext 4, GE Healthcare 2015). Lesion location on PET imagery was confirmed by fusing PET and MRI brain axial datasets using the Neuro Registration application of the Volume Viewer software package.

For quantitative assessment, tracer VOI-based measurements of FDG uptake were quantified using SUV parameters normalized to patients’ body weight, including SUV_max_ the maximum voxel value in the VOI; SUV_peak_ the highest average SUV in any 1 cc spherical subregion of the VOI (automatically identified by the software); and SUV_mean_ the average SUV of all voxels in the VOI. Metabolic tumor volume (MTV) (volume encompassed by a 42% isocontour around the voxel with the highest PET uptake) and total lesion glycolysis (TLG) (calculated by multiplying MTV by SUV_mean_) were also determined. Brain tumor volume was quantified by adapting the semiautomated delineation technique and thresholding value reported by colleagues at our institution for body PET/CT.^18^ Additional VOIs were then drawn in the contralateral normal white matter at the centrum semiovale. Comparative lesion uptake was quantified using target-to-normal white matter (TNR-WM) ratios defined as SUV_max_(lesion)/SUV_mean_ (white matter).

For qualitative assessment, lesions were considered non-FDG-avid if tracer uptake was less than, equivalent to, or only mildly higher than normal white matter uptake, by subjective visual analysis of PET imagery (these categories of uptake were combined due to small sample size); lesions with higher tracer uptake were considered FDG-avid, noting that viable GBM is consistently reported as more FDG-avid than white matter in other clinical settings.^19^ These determinations were made independently by 2 nuclear medicine physicians, with a 97% concordance rate. The single discordant case was discussed, and a consensus was reached.

### Statistics

Kaplan–Meier survival curves were generated to examine the OS experience of the study cohort. Log-rank test was used to compare OS between FDG-avid and non-avid patients. Univariable Cox proportional hazards regression was used to examine associations between FDG PET parameters and OS, with a false discovery rate (FDR) adjustment applied. Multivariable Cox proportional hazards regression was used to analyze the association between FDG avidity or SUV_max_ and OS while controlling for other patient and tumor characteristics (age, Karnovsky Performance Status [KPS], and *MGMT* promoter methylation status). A significance level of .05 was used throughout.

Maximally selected rank statistics were used to identify potentially “optimal” cutoff values for SUV_max_. We considered nine cutoff values (representing the 10th through 90th percentile values) for each variable. Kaplan–Meier survival curves and log-rank tests were used to explore associations between these cutoff values and OS. We adjusted the log-rank *P*-values to account for the testing of multiple cutoff candidates.^20^ All statistical computations were performed, and all output was generated using SAS Software Version 9.4 (The SAS Institute).

## Results

### Patient Baseline Characteristics

Thirty-one patients with recurrent GBM were included in the study (22 males and 9 females, age 36–81 years, median 54 years) (Table 1). Median KPS was 80, with a range of 40–100. Four tumors (12.9%) showed *MGMT* promoter methylation. Median time from initiation of bevacizumab treatment to FDG PET was 4.3 months, with a range of 0–17.4 months. This is in keeping with the median PFS for bevacizumab documented in the literature. A more detailed description of sample characteristics for each patient, including the RANO criteria for the progressive disease that were met, is included in Supplementary Table 1.

**Table 1.** Patient Clinical and Radiographic Characteristics

		All Patients	FDG Non-avid	FDG Avid
		*N* (%)	*N* (%)	*N* (%)
*Clinical characteristics*				
Sample size		31	7 (22.6)	24 (77.4)
Age at diagnosis	Median (range)	54 (36–81)	53 (36–64)	54 (44–81)
Sex	Male	22 (71)	5 (71.4)	17 (70.8)
	Female	9 (29)	2 (28.6)	7 (29.2)
KPS	Median (range)	80 (40–100)	80 (60–90)	80 (40–100)
*MGMT* status	Methylated	4 (12.9)	0 (0)	4 (16.7)
	Unmethylated	20 (64.5)	5 (71.4)	15 (62.5)
	Unknown	7 (22.6)	2 (28.6)	5 (20.8)
No. of prior recurrences	Median (range)	1 (0–4)	1 (0–3)	1 (0–4)
Time from most recent radiation (months)	Median (range)	12.0 (1.3–51.5)	6.3 (1.4–16.8)	12.6 (1.3–51.5)
Time from bevacizumab initiation (months)	Median (range)	4.3 (0–17.4)	4.3 (0.1–17.4)	4.7 (0–12.7)
*PET parameters*				
SUV_max_	Median (range)	9.7 (2.7–52.8)	5.5 (2.7–8.7)	11.7 (6.8–52.8)
SUV_peak_	Median (range)	7.4 (4.0–35.3)	5.2 (4.0–6.2)	7.8 (5.0–35.3)
MTV	Median (range)	5.1 (0.2–47.0)	3.2 (0.2–11.7)	7.2 (1.1–47.0)
TLG	Median (range)	26.8 (0.3–469.1)	13.3 (0.3–58.4)	66.0 (7.7–469.1)
TNR-WM	Median (range)	3.4 (1.2–17.6)	2.1 (1.2–3.0)	3.9 (2.4–17.6)

### FDG PET Measurements

FDG PET measurements are summarized in Table 1. The median SUV_max_ for the cohort was 9.7 (range, 2.7–52.8). The median SUV_peak_ was 7.4 (range, 4.0–35.3), with non-measurable SUV_peak_ in 4 patients due to small lesion sizes. To ensure intra- and inter-individual comparability, SUV_max_ was also normalized to FDG avidity in contralateral white matter (TNR-WM), yielding a median of 3.4 (range, 1.2–17.6). The median MTV was 5.1 (range, 0.2–47.0) and median TLG was 26.8 (range, 0.3–469.1). Boxplots displaying the spread of individual data points for each PET parameter are included in Supplementary Figure 1.

Qualitative assessment of FDG PET scans was also performed, and patient lesions were divided into FDG non-avid and FDG avid subgroups. Seven patients (22.6%) were categorized as non-avid and 24 patients (77.4%) were categorized as avid. These patients had similar clinical characteristics, including median age at diagnosis, gender, KPS, number of prior recurrences, and time from initiation of bevacizumab therapy (Table 1). All 4 *MGMT*-methylated tumors were FDG avid, despite the increased incidence of pseudoprogression described in these tumors.^21^

### Association With OS

**Figure 1.**
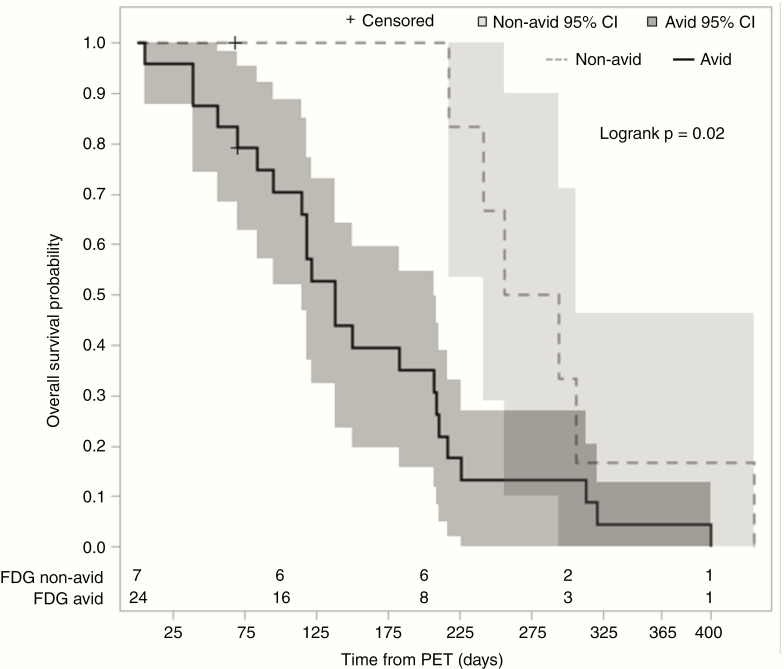
Qualitative FDG avidity during bevacizumab treatment predicts overall survival. Kaplan–Meier survival curves for patients separated into FDG-avid (solid line, *n* = 24) and FGD-non-avid (dotted line, *n* = 7), with 95% confidence intervals shaded. The number of patients at risk at 100-day intervals are delineated at the bottom.

**Figure 2.**
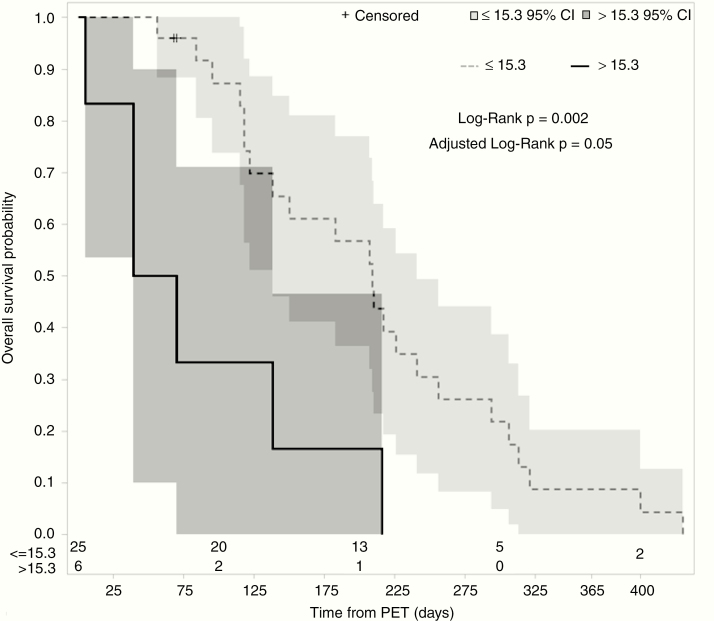
SUV_max_ during bevacizumab treatment predicts overall survival. Kaplan–Meier survival curves for patients stratified by SUV_max_: SUV_max_ more than 15.3 (solid line, *n* = 6) and SUV_max_ not more than 15.3 (dotted line, *n* = 25), with 95% confidence intervals shaded. The number of patients at risk at 100-day intervals are delineated at the bottom.

Median OS from the time of PET scan for all patients was 6.0 months (95% confidence interval [CI] 3.9–7.1). When stratified by qualitative FDG avidity, the non-avid cohort had a longer median OS of 9.0 months as compared with the avid cohort at 4.5 months. Log-rank analysis demonstrated a difference in OS between the 2 groups (*P* = .02, Figure 1). Univariable Cox regression analysis demonstrated a significant association of qualitative FDG avidity with inferior OS (hazard ratio [HR] 2.97; 95% CI 1.10–7.99; *P* = .03) (Table 2). Higher SUV_max_, SUV_peak_, and TNR-WM were also associated with an inferior OS on univariable Cox regression analysis (*P* < .001), as was TLG (*P* = .009) (Table 2). These associations remained significant after FDR adjustment. MTV, however, was not significantly associated with OS (*P* = .59). In multivariable Cox regression analysis including FDG avidity, SUV_max_, age at diagnosis, KPS, and MGMT status, only FDG avidity (HR 3.49, 95% CI 1.02–11.92, *P* = .046) and SUV_max_ (HR 1.05, 95% CI 1.00–1.11, *P* = .048) were significantly associated with OS (Table 3). We also sought to determine an optimal cutoff for SUV_max_ that maximized difference in OS between the 2 groups using maximally selected rank statistics. The optimal cutoff was 15.3, with a median OS of 6.9 months (95% CI 4.5–7.9 months) in patients with SUV_max_ not more than 15.3 as compared with 1.8 months (95% CI 1.3–4.5 months) in patients with SUV_max_ more than 15.3 (adjusted *P* = .048, Figure 2).

**Table 2.** Univariable Cox Proportional Hazards Regression Analysis of PET Parameters With OS

Variable	HR (95% CI)	*P*
SUV_max_	1.080 (1.034–1.128)	<.001*
SUV_peak_	1.115 (1.046–1.189)	<.001*
MTV	1.008 (0.979–1.039)	.59
TLG	1.005 (1.001–1.008)	.009*
TNR-WM	1.308 (1.135–1.507)	<.001*
FDG avidity		
Non-avid	Ref.	
Avid	2.968 (1.102–7.989)	.03*

*Significant after FDR adjustment.

**Table 3.** Multivariable Cox Proportional Hazards Regression Analysis of FDG Avidity and SUV_max_ With OS

Variable		HR (95% CI)	*P*
FDG avidity	Non-avid	Ref.	
	Avid	3.49 (1.02–11.92)	.046
Age at diagnosis		0.98 (0.92–1.04)	.5
SUV_max_		1.05 (1.00–1.11)	.048
KPS		0.99 (0.96–1.02)	.62
MGMT	Unmethylated	Ref.	
	Methylated	0.53 (0.13–2.15)	.37
	Unknown	2.71 (0.78–9.34)	.11

### Histopathological Correlation

**Figure 3.**
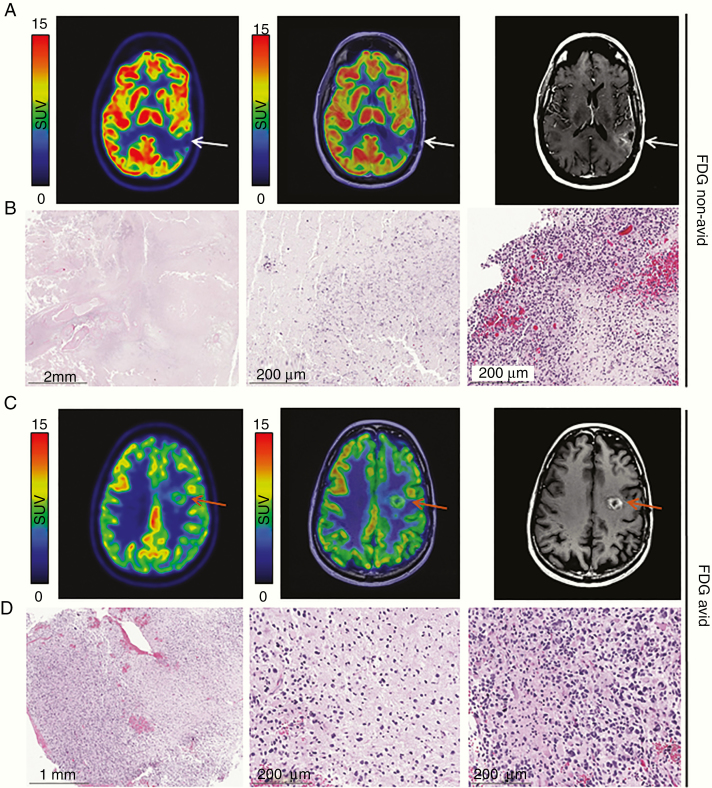
A histopathologic correlate of FDG non-avid and FDG avid lesions. (A) Axial PET (*left)* and PET/MR (*middle*) of an FDG non-avid lesion demonstrate no distinct uptake in the left parietal region (white arrow) which enhances on post-contrast T1-weighted MRI (*right*). (B) On H&E, the FDG non-avid specimen consists almost entirely of necrotic tissue (*left*), with microcalcification, apoptotic debris (*middle*), and residual malignant glioma *(right)* focally present. (C) Axial PET (*left*) and PET/MR (*middle*) of an FDG avid lesion demonstrate focal uptake in the left frontal region (orange arrow) which enhances on post-contrast T1-weighted MRI *(right).* (D) On H&E, the FDG avid specimen contains minimal necrosis, consisting of predominately viable appearing tumor *(left)* in both highly cellular regions *(right*) and areas of infiltrating tumor *(middle).*

Seven patients (22.6%) had tissue available for histopathologic evaluation within 3 months of FDG PET, either due to re-resection (*n* = 6) or autopsy (*n* = 1). Of these patients, 1 (14.3%) had been categorized as non-avid and 6 (85.7%) as avid. There was strong concordance between the histopathologic findings and FDG avidity in these patients: the one non-avid lesion demonstrated predominantly necrosis with minute foci of residual malignant glioma (Figure 3A and B) while conversely a representative avid lesion showed a predominantly viable tumor with minimal necrosis (Figure 3C and D). The additional 5 avid lesions were also reported as being comprised of predominantly tumors with varying amounts of necrosis.

## Discussion

Prognostication of recurrent GBM in the setting of chronic antiangiogenic therapy remains a pervasive and clinically relevant challenge.^22^ The present study demonstrates an important association between FDG PET avidity and OS from the time of concern for progression in recurrent GBM patients on bevacizumab therapy, suggesting its potential utility as an adjunct tool in assessing prognosis in this setting. Qualitative lesion analysis showed that patients with non-avid lesions had a median OS twice that of patients with avid lesions, with a univariable HR of 2.97. Additionally, increased quantitative PET parameters SUV_max_, SUV_peak_, TLG, and TNR-WM were also significantly associated with inferior OS. The association of FDG avidity and SUV_max_ with inferior OS was confirmed in a multivariable analysis, though it should be noted that the constraint of our small sample size may impact the reliability of this assessment. Conversely, MTV showed no significant association with OS. This is in agreement with a recent systematic review and meta-analysis assessing the prognostic value of PET parameters in glioma.^23^ It is important to note that accurate determination of MTV in brain tumors is complicated by the technical challenge of delineating tumor boundaries due to the high physiologic uptake of FDG in gray matter. We adapted a semiautomated approach to determine tumor volume to address this issue. An SUV_max_ threshold that optimally stratified patients by OS was identified. This may contribute to more broad generalizability of our findings, as SUV_max_ is the most commonly used quantitative PET parameter. Importantly, histopathological correlation when available was concordant with FDG avidity in every sample. This correlation reinforces the accuracy of FDG avidity as a possible indicator of viable tumor in the setting of bevacizumab therapy.

Recent studies exploring the role of PET imaging in tumor recurrence have focused on amino acid tracers, most commonly [^11^C]-methyl-methionine (MET), O-(2-[^18^F]-fluoroethyl)-l-tyrosine (FET), and 3,4-dihydroxy-6-[^18^F]-fluoro-L-phenylalanine (FDOPA).^19,24^ Such tracers have lower background uptake in normal brain tissue than FDG and so are thought to have enhanced ability to reliably identify tumor tissue. A previous study by Harris et al.^25^ demonstrated that FET and FDOPA uptake metrics were correlated with 3-month PFS and 6-month OS in GBM patients treated with bevacizumab. However, only FDOPA parametric response maps were significantly predictive of OS. A similar study by Beppu et al.^26^ investigated the utility of MET PET in this setting and found MET uptake at 8 weeks to be predictive of PFS. However, PFS is a surrogate outcome known to be disconnected from OS in the setting of bevacizumab use, and this study did not include an analysis of OS. Furthermore, both studies performed PET imaging within 2 months of bevacizumab initiation and so are unable to comment on the role of PET imaging as a biomarker in the setting of prolonged bevacizumab use. Finally, none of these amino acid tracers are FDA-approved for use in brain tumors,^27^ which limits their widespread use in the United States.

A few prior studies have suggested the potential prognostic utility of FDG PET in the setting of antiangiogenic therapy in malignant gliomas. FDG avidity at 4 weeks (as measured by SUV_median_) was predictive of worse OS in a study of bevacizumab monotherapy for recurrent anaplastic glioma.^28^ In recurrent high-grade glioma patients treated with bevacizumab and irinotecan, FDG uptake within 6 weeks of treatment initiation was the most powerful predictor of PFS and OS of all variables tested in multivariate analysis.^29^ The authors were also able to establish objective cutoffs, with SUV_max_ more than 7 and tumor-to-normal contralateral brain ratio more than 1.348 correlated with decreased OS in both univariate and multivariate analyses. While these results suggest a prognostic value of early FDG PET in this patient population, they do not inform the utility of FDG PET performed after prolonged antiangiogenic therapy. In an exploratory imaging analysis, Omuro et al.^16^ demonstrated that FDG avidity at 6 months was associated with worse OS. However, this phase II clinical trial enrolled newly diagnosed GBM patients and initiated bevacizumab as part of upfront therapy, in stark contrast to its current clinical use primarily at relapse. To our knowledge, the present study is the first evaluation of the prognostic value of FDG PET including recurrent GBM patients on prolonged antiangiogenic therapy.

There are several potential limitations to the current study. First, it is based on retrospective data, and as such there is inevitable variability in several patient characteristics, such as the duration of bevacizumab therapy prior to PET scan. Second, the sample size of 31 with only 7 FDG non-avid patients is relatively small which limits the power of our statistical modeling, though it is similar to the overall sample size of the prospective PET studies referenced above. Finally, this is a single-center study and thus may not capture the heterogeneity that would be seen in a multicenter study. Our results should be confirmed with a prospective study in a larger, more consistently defined patient cohort.

In conclusion, FDG PET is a promising prognostic imaging tool in recurrent GBM patients on prolonged antiangiogenic therapy and may serve as an adjunct to MRI for prognostication.

## Supplementary Material

vdaa050_suppl_Supplementary_Tables

vdaa050_suppl_Supplementary_Figure_1
