# Supplementary material for: Value of [18F]-FDG positron emission tomography in patients with recurrent glioblastoma receiving bevacizumab
Source: Neurooncol Adv. 2020 Apr 15;2(1):vdaa050. doi: 10.1093/noajnl/vdaa050 (PMC7236386; doi:10.1093/noajnl/vdaa050)
Supplement: vdaa050_suppl_Supplementary_Tables [file vdaa050_suppl_supplementary_tables.docx]

**Supplemental Table 1: Sample characteristics for each patient.**

| **Patient** | **Age at Diagnosis** | **MGMT status** | **# of Recurrences** | **Time from RT (mon)** | **KPS at PET** | **FDG Avidity** | **SUV_max_** | **RANO Criteria for PD** |
| --- | --- | --- | --- | --- | --- | --- | --- | --- |
| 1 | 56 | Unmethylated | 2 | 7.2 | 60 | Avid | 13.3 | T1-Gd |
| 2 | 53 | Unknown | 2 | 7.7 | 70 | Avid | 12.3 | T1-Gd |
| 3 | 51 | Unmethylated | 3 | 15.7 | 80 | Avid | 23.88 | T1-Gd |
| 4 | 52 | Unmethylated | 1 | 1.4 | 70 | Non-avid | 5.53 | T1-Gd |
| 5 | 44 | Unmethylated | 2 | 13.4 | 80 | Avid | 30.66 | T1-Gd |
| 6 | 41 | Unmethylated | 2 | 17.0 | 70 | Non-avid | 8.7 | T1-Gd |
| 7 | 56 | Unmethylated | 5 | 48.8 | 80 | Avid | 9.08 | T1-Gd |
| 8 | 54 | Unmethylated | 4 | 21.0 | 80 | Avid | 8.71 | T1-Gd |
| 9 | 60 | Unmethylated | 2 | 16.3 | 90 | Non-avid | 5.4 | T1-Gd |
| 10 | 51 | Unmethylated | 4 | 42.5 | 70 | Avid | 10.3 | T1-Gd |
| 11 | 51 | Unmethylated | 3 | 10.8 | 60 | Avid | 11.1 | T1-Gd |
| 12 | 50 | Unmethylated | 2 | 6.2 | 80 | Avid | 7.7 | T1-Gd |
| 13 | 46 | Methylated | 3 | 23.1 | 80 | Avid | 15.78 | T1-Gd |
| 14 | 44 | Unknown | 3 | 15.7 | 80 | Avid | 25.2 | T1-Gd |
| 15 | 56 | Unmethylated | 2 | 18.5 | 90 | Avid | 26.3 | T1-Gd |
| 16 | 36 | Unmethylated | 4 | 13.6 | 80 | Non-avid | 6.8 | T1-Gd |
| 17 | 77 | Unknown | 1 | 1.3 | 70 | Avid | 9.6 | T1-Gd |
| 18 | 60 | Unmethylated | 2 | 12.2 | 50 | Avid | 15.3 | T1-Gd |
| 19 | 53 | Unmethylated | 3 | 4.5 | 70 | Non-avid | 5.31 | T1-Gd |
| 20 | 64 | Unknown | 2 | 2.4 | 70 | Non-avid | 7.5 | T1-Gd |
| 21 | 44 | Methylated | 3 | 51.5 | 100 | Avid | 8.1 | T1-Gd |
| 22 | 61 | Unmethylated | 2 | 10.5 | 40 | Avid | 8.4 | T1-Gd |
| 23 | 54 | Unmethylated | 5 | 24.7 | 90 | Avid | 11.9 | FLAIR |
| 24 | 52 | Methylated | 4 | 4.6 | 80 | Avid | 6.81 | T1-Gd |
| 25 | 53 | Unmethylated | 3 | 13.8 | 70 | Avid | 9.65 | T1-Gd |
| 26 | 58 | Unmethylated | 2 | 5.1 | 100 | Avid | 13.7 | T1-Gd |
| 27 | 81 | Unknown | 2 | 5.6 | 90 | Avid | 11.57 | T1-Gd |
| 28 | 61 | Unknown | 1 | 6.4 | 50 | Non-avid | 2.74 | FLAIR |
| 29 | 56 | Methylated | 2 | 14.3 | 90 | Avid | 8.6 | T1-Gd |
| 30 | 62 | Unmethylated | 3 | 4.0 | 80 | Avid | 14.9 | T1-Gd |
| 31 | 62 | Unknown | 2 | 4.7 | 60 | Avid | 52.8 | T1-Gd |

**SUPPLEMENTAL FIGURE CAPTIONS:**

**Supplemental Figure 1. Spread of PET parameter data points.**

A) Box and whisker plots of all data points for SUV_max_, SUV_peak_, MTV and TNR-WM.
B) Box and whisker plot of all data points for TLG (shown on separate plot given disparate *y* axis range). Whiskers represent 1.5 x IQR (interquartile range).
