# Supplementary figures and images for: Value of [18F]-FDG positron emission tomography in patients with recurrent glioblastoma receiving bevacizumab
Source: Neurooncol Adv. 2020 Apr 15;2(1):vdaa050. doi: 10.1093/noajnl/vdaa050 (PMC7236386; doi:10.1093/noajnl/vdaa050)

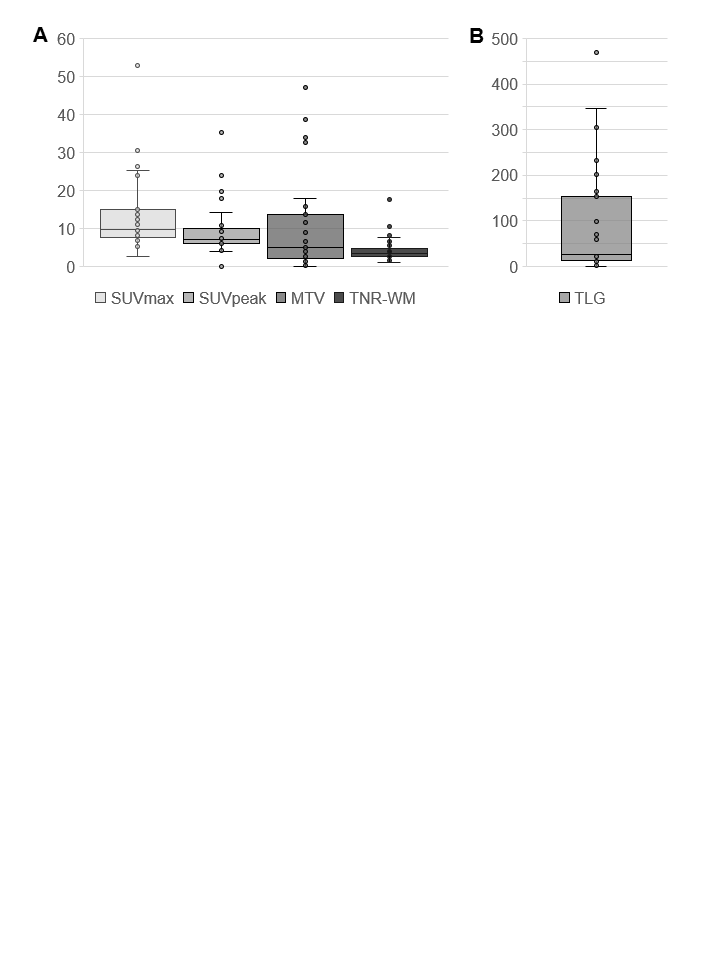

Supplement: vdaa050_suppl_Supplementary_Figure_1 [file vdaa050_suppl_supplementary_figure_1.png]
